# Effect of Different Insulin Therapies on Obstetric-Fetal Outcomes

**DOI:** 10.1038/s41598-019-54164-4

**Published:** 2019-11-27

**Authors:** Cristina López-Tinoco, José Luis Jiménez-Blázquez, Laura Larrán-Escandón, María del Mar Roca-Rodríguez, Fernando Bugatto, Manuel Aguilar-Diosdado

**Affiliations:** 1 Department of Endocrinology, Puerta del Mar Hospital Cádiz/ Department of Medicine, University of Cádiz, Av. Ana de Viya 21 CP:11009 Cádiz/Dr. Marañón, 3 CP: 11002 Cádiz, Spain; 2 Department of Medicine, University of Cádiz, Dr. Marañón, 3 CP: 11002 Cádiz, Spain; 3 Department of Endocrinology, Puerta del Mar Hospital, Av. Ana de Viya 21, CP: 11009 Cádiz, Spain; 4 Department of Obstetrics and Gynaecology, Puerta del Mar Hospital, Cádiz, Av. Ana de Viya 21, CP: 11009 Cádiz, Spain

**Keywords:** Endocrinology, Endocrine system and metabolic diseases, Diabetes, Diabetes complications

## Abstract

To evaluate the effectiveness of the different insulin therapies on obstetrics-fetal outcomes in women with pregestational diabetes mellitus. We enrolled 147 pregnant women with pre-existing type 1 or 2 diabetes mellitus. Clinical and biochemical parameters were analysed in relation to obstetric and fetal outcomes. 14.2% received treatment with Neutral Protamine Hagedorn insulin and short-acting insulin analogues; 19% with premixed human insulin; 40.1% with insulin glargine and lispro, 6.2% with detemir and aspart and 20% with continuous subcutaneous insulin infusion. All 5 types of treatment achieved a reduction of the mean HbA1c during pregnancy (p = 0.01). Pre-pregnancy care was carried out for 48% of patients. We found no statistically significant differences between the different insulin therapies and the obstetric-fetal outcomes. In conclusión, the different insulin therapies used in patients with pregestational diabetes mellitus does not seem to affect obstetric-fetal outcomes.

## Introduction

Diabetes mellitus is one of the most frequent metabolic complications of gestation and it is associated with an increased risk of maternal and fetal morbidity and mortality^1^. Approximately 1% of all pregnant women present pregestational diabetes mellitus (PGDM), however, this prevalence is increasing^2^. It is widely acknowledged that pre-pregnancy care (PPC) is beneficial for women with PGDM to minimize risks in the fetus and the mother^3^.

Women with PGDM present worse results than pregnant women without diabetes mellitus, with an increase in the rate of congenital malformations, preeclampsia, preterm birth, perinatal mortality, macrosomia^4,5^ and worsening of retinopathy and diabetic nephropathy^6^. Therefore, it is essential to achieve an optimum glycaemic control before and during gestation^1^.

Nowadays, insulin is the treatment of choice for PGDM, however, there is no clear evidence of a difference between regular human insulin and other types of insulin in gestational diabetes^7^. In fact, alterations in the placenta and umbilical vessels in the fetus of women with gestational diabetes treated with insulin have been seen^8^.

Neutral Protamine Hagedorn (NPH) insulin is recommended. Several studies support the safety and efficacy of insulin analogues in pregnancy^9^. The short-acting insulin analogues (lispro and aspart) are comparable in immunogenicity to rapid human insulin and they are not teratogenic. And they also achieve the objectives of postprandial control with lower risk of hypoglycaemia^10^. The long-acting insulin analogues (detemir and glargine) improve the fasting plasma glucose and glucose fluctuations without an increased incidence of nocturnal hypoglycaemia^9^ and they have not demonstrated significant adverse effects on outcomes of the pregnancy^9,10^. In fact, since 2012, the Food and Drug Administration (FDA) has reclassified basal insulins from category “C” to “B” and so they can be used in pregnancy^9^.

In women with type 1 PGDM, if adequate glycaemic control is not achieved with the use of multiple doses of insulin (MDI) in bolus-basal regimen (long-acting insulin analogues plus short-acting insulin analogues at each meal), the use of continuous subcutaneous insulin infusion (CSII) is recommended^11^. In women with type 2 PGDM who receive oral antidiabetic treatment, they should be treated with insulin therapy during pregnancy. Ideally, insulin therapy should be initiated during the preconception period^1^.

Thus, it is important to know whether the different insulin therapies used in patients with PGDM has an impact on obstetrics-fetal outcomes, and whether the establishment of therapeutic and preventive strategies can reduce them.

The aim of the present study was to evaluate the effectiveness of the different insulin therapies on obstetrics-fetal outcomes and to assess the impact on maternal metabolic outcomes in relation to the PPC.

## Study Design

### Study population

We enrolled 147 pregnant women for this study from among subjects who were attending the Endocrinology and Pregnancy Clinic of Puerta del Mar University Hospital (Cádiz, Spain) from February 2007 until June 2017. The women included had a singleton gestation, pre-existing type 1 or 2 DM, clinical follow-up from the first trimester of gestation until delivery. The candidates who fulfilled the selection criteria were informed of the characteristics and objectives of the study before they signed specially-designed informed consent forms to participate in the study.

Study subjects included 113 women with type 1 PGDM and 34 with type 2 PGDM. Both groups of women were comparable; there was no significant difference with respect to BMI. They had the same modality of treatment from the moment of inclusion of the study until delivery, only the dosage had been modified. Patients with type 2 PGDM received insulin treatment with short-acting insulin analogues (lispro and aspart) and NPH insulin or premixed human insulin, consisting of rapid-acting and intermediate-acting insulin analogues with different fixed-ratio mixtures of insulin preparations (for example Humalog Mix 25/50 or Novomix 30/50/70), administered two or three times daily; or MDI; three injections of rapid-acting insulin analogues before their three main meals and one injection of insulin (detemir or glargine) in the morning or and in the evening (bolus basal regimen). Patients with type 1 PGDM received treatment with MDI in bolus-basal regimen or CSII; insulin infusion bolus doses of rapid-acting insulin analogues were given immediately prior to the three main meals, while the basal infusion of insulin was adjusted hour by hour, according to the glycaemic targets for fasting. All women in the continuous subcutaneous insulin infusion group started the treatment with pumps before pregnancy. Our clinic’s dietician prescribed individualised nutritional therapy.

Prenatal visits were performed every week until delivery. HbA1c was assessed every 4 to 6 weeks. Each woman had an ultrasound scan once a trimester (at about 12, 20, 34 weeks), according to the Spanish guidelines for pregnancy monitoring. In addition, an ultrasound scan at 32 weeks was performed to assess fetal growth.

Maternal characteristics collected included: age at conception, prepregnancy body mass index (BMI) and at the end of pregnancy, weight gain in pregnancy, duration of diabetes and microvascular complications at conception (such as retinopathy, nephropathy, neuropathy), preconception care or not. Maternal metabolic data collected were: prepregnancy HbA1c, enrolment and at the end of pregnancy, insulin requirements at the beginning and end of pregnancy. Maternal outcomes included progression of microvascular complications (retinography and albumin/creatinine ratio were performed in each trimester of pregnancy), rate of non-elective Caesarean section, elective Caesarean section, vaginal delivery, abortion and fetal death.

Fetal outcomes included: abdominal circumference of the fetus measured by ultrasound scan at week 32 of pregnancy, gestational age at birth, birth weight, newborn weight percentile, rate of preterm birth (before 37 weeks), and macrosomia and APGAR score at 5 minutes after birth.

All biochemical parameters were analysed at the clinical laboratory of our hospital. Glucose was determined in venous blood using the biochemical system Modular DPD (Roche Diagnostics, Switzerland). HbA1c was measured in the Cobas Integra 700 autoanalyser (Roche Diagnostics, Switzerland) using an immunoturbidimetric method for completely haemolysed, anti-coagulated blood. The albumin/creatinine ratio was determined using an immunoturbidimetric test in the Cobas Integra 700 system (Roche Diagnostics, Switzerland).

### Ethical approval

The study protocol was approved by the ethical committee of the University Hospital Puerta del Mar and followed the guidelines of the Helsinki protocol.

### Statistical analysis

Descriptive statistics included mean, median, standard deviation and ranges for quantitative variables, and percentages for qualitative variables. Associations between categorical variables-of-interest and their dependent and independent variables were determined by the Mantel-Henszel χ^2^ test with the Yates correction, or the Fisher test if the variable contained <5 measurements. Parametric variables were compared using the Student *t*-test, and one-way analysis of variance (ANOVA). We used the Bonferone test, if the results were statistically significant. Non-parametric variables were evaluated with the Mann-Whitney test. Correlations between variables were evaluated using Spearman’s rank correlation coefficient. A level of p < 0.05 was accepted as being statistically significant. Statistical analyses were with the Statistical Package for Social Sciences (SPSS 21.0 for Windows).

## Results

Demographic, clinical and laboratory variables of the studied population are summarised in Table 1.

**Table 1:** Demographic, clinical and laboratory variables in type 1 PGDM and type 2 PGDM.

	Type 1 PGDM	Type 2 PGDM	p
Maternal age; years	30.3 +/− 4.9	31.2 +/− 4.7	0.3
Duration of diabetes; years	16.1 +/− 6.6	3.9 +/− 2.1	0.001
Pre-pregnancy care; N(%)	40 (35%)	9 (26%)	0.01
**Micro-vascular complications at conception;**			
Retinopathy; N(%)	41 (40%)	2 (6%)	0.001
Nephropathy; N%	21(18%)	0	0.05
Pre-pregnancy body mass index (BMI); kg/m^2^	24.3 +/− 2.8	25.7 +/− 3.6	0.06
BMI at the end of pregnancy; kg/m^2^	30.1 +/− 2.6	30.4 +/− 2.7	0.6
Weight gain in pregnancy; Kg	3.9 +/− 1.3	4.6 +/− 1.2	0.09
Pre-pregnancy HbA1c; %	7.6 +/− 1.4	7.0 +/− 1.6	0.08
Mean HbA1c in pregnancy; %	6.7 +/− 1.0	6.1 +/− 0.8	0.05
Insulin requirements at the beginning of pregnancy; UI/Kg	0.7 +/− 0.2	0.4 +/− 0.2	0.01
Insulin requirements at the end of pregnancy; UI/Kg	0.8 +/− 0.2	0.6 +/− 0.2	0.01

Values are given as mean +/− SD or number of subjects (n) with percentage in parenthesis.

There were 5 types of insulin therapy; 14.2% received treatment with NPH insulin and short-acting insulin analogues; 19% with premixed human insulin; 40.1% with insulin glargine and lispro, 6.2% with detemir and aspart and 20.5% with continuous subcutaneous insulin infusion.

Ten women (7,3%) had miscarriages. Nine were type 1 PGDM; all of them had 10 or more years duration of diabetes and poor metabolic control prior to gestation, with HbA1c of 8%. Most of them were treated with glargine and lispro except for one who was treated with NPH insulin and lispro. Only one patient with type 2 PGDM had a pre-pregnancy HbA1c of 12% and was treated with premix insulin. Five women (3.6%) had fetal death. Three were type 1 PGDM, one of them had twenty years duration of diabetes, a pre-pregnancy HbA1c of 10% and mean HbA1c of 8.67%, and was treated with continuous subcutaneous insulin infusion. The other two fetal deaths had a mean HbA1c of 7.53% and were treated with glargine and lispro. A fetal brain malformation was observed in one of them. In another the fetal death was due to the development in the mother of a diabetic ketoacidosis and in the last case it was not possible to identify the cause of fetal death. The other patients with type 2 PGDM had 3 years duration of diabetes, a pre-pregnancy HbA1c of 8% and mean HbA1c of 7% and were treated with NPH insulin and lispro. One fetal death was due to multiple anomalies and the other was unexplained.

To investigate the relationship between obstetrics and fetal outcomes and the pre-pregnancy and mean HbA1c, we observed that birth weight, weight percentile, end of pregnancy route and gestational age at birth were significantly related to pre-pregnancy and mean HbA1c during pregnancy. All data are summarized in Table 2.

**Table 2:** Relationship between obstetric and fetal outcomes and pre-pregnancy and mean HbA1c in pregnancy.

Obstetric and fetal outcomes	Pre-pregnancy HbA1c	Mean HbA1c in pregnancy
Fetal abdominal circumference by ultrasound scan at week 32 of pregnancy	P = 0.5	P = 0.4
- High range (>298 mm)	7.7 +/− 1.7	6.5 +/− 0.8
- Normal range (<298 mm)	6.9 +/− 1.4	6.4 +/− 0.9
Birth weight	P = 0.2	P = 0.04
− <2500 g	7.4 +/− 1.6	6.2 +/− 0.8
− 2500–3999 g	7.1 +/− 1.5	6.3 +/− 0.9
− >4000 g	7.8 +/− 1.7	6.7 +/− 0.9
Weight percentile	P = 0.007	P = 0.001
- Normal weight	6.7 +/− 1.6	6.1 +/− 0.8
- Macrosomia	7.2 +/− 1.6	6.7 +/− 0.9
End of Pregnancy Route	P = 0.01	P = 0.001
- Vaginal birth	6.8 +/−1.4	6.1 +/− 0.7
- Elective Cesarean	7.0 +/− 1.5	6.5 +/− 1.0
- Non- Elective Cesarean	7.7 +/− 1.6	6.5 +/− 0.8
- Miscarriage	8.2 +/− 1.8	7.9 +/− 1.9
- Fetal death	8.5 +/− 1.1	7.5 +/− 0.7
Gestational age at birth	P = 0.01	P = 0.001
- Preterm birth	7.8 +/− 1.5	7.1 +/− 1.3
- Birth to term	7.0 +/− 1.6	6.2 +/− 0.8

The incidence of preterm birth was higher in women with a poor periconceptional HbA1c level. The 18% refers to spontaneous preterm birth. Most of them were medically indicated preterm birth; Caesarean in 73% of the cases of which 71% were emergency Caesarean - of which two were due to the development of severe preeclampsia, one was due to diabetic ketoacidosis in the mother and seven for induced birth following fetal macrosomia.

The association of different insulin therapies in PGDM, pre-pregnancy and mean HbA1c during pregnancy is shown in Table 3.

**Table 3:** Effectiveness of different insulin therapies on HbA1c levels.

Insulin therapies	Pre-pregnancy HbA1c%	Mean HbA1c in pregnancy%
**a) Type 1 PGDM**		
NPH and short-acting insulin analogues	7.8 +/− 1.1	6.9 +/− 0.9*
Glargine and Lispro	7.6 +/− 1.4	6.6 +/− 0.7*
Detemir and Aspart	8.3 +/− 0.7	7.04 +/− 0.7*
CSII	7.4 +/− 1.3	6.6 +/− 0.8*
**b) Type 2 PGDM**		
NPH and short-acting insulin analogues	7.8 +/− 1.1	6.9 +/− 0.9*
Premixed human insulin	6.2 +/− 1.4	5.6 +/− 0.5*
Glargine and Lispro	7.4 +/− 0.8	6.8 +/− 0.3*
Detemir and Aspart	7.1 +/− 1.1	6.1 +/− 0.8*

NPH; Neutral Protamine Hagedorn, CSII; continuous subcutaneous insulin infusion.

*p < 0.05.

NPH; Neutral Protamine Hagedorn,

*p < 0.05.

The relation between different insulin therapies used and the obstetric and fetal outcomes were analysed, no statistically significant differences were found (Table 4).

**Table 4:** Obstetrics and fetal outcomes and different insulin therapies used in relation to good and poor glycaemic control.

Obstetrics and fetal outcomes/ Insulin therapies	Good glycaemic control (Hba1c < 7%)	Poor glycaemic control (Hba1c > 7%)	p
**Gestational age, weeks;**			
Glargine and Lispro	36.5 +/− 8.6	36.1 +/− 2.5	0.4
CSII	36.3 +/− 2.4	35.6 +/− 3.8	0.6
Premixed human insulin	38.1 +/− 2.2	37.8 +/− 0.4	0.7
Detemir and Aspart	38 +/− 0.1	38.1 +/−1.2	0.9
NPH and insulin analogue	38.1 +/− 8.6	36.1 +/−8.6	0.8
**Macrosomia N (%);**			
Glargine and Lispro	0	1(66%)	0.7
CSII	1(25%)	3(75%)	0.08
Premixed human insulin	0	1(66%)	0.3
Detemir and Aspart	0	0	0.8
NPH and insulin analogue	0	1(50%)	0.9
**Fetal abdominal circumference by ultrasound scan at week 32 of pregnancy; mm**			
Glargine and Lispro	277 +/−29	308 +/−19	0.4
CSII	287 +/−20	300 +/−26	0.1
Premixed human insulin	254 +/−76	295 +/−33	0.09
Detemir and Aspart	348 +/−10	352 +/−26	0.1
NPH and insulin analogue	302 +/−8	307 +/−2	0.5
**Birth weight; gr;**			
Glargine and Lispro	3271 +/−853	3641 +/−477	0.09
CSII	3045 +/−953	3496 +/−567	0.2
Premixed human insulin	3149 +/−436	3478 +/−675	0.3
Detemir and Aspart	3557 +/−134	3399 +/−239	0.1
NPH and insulin analogue	3762 +/−553	3552 +/−597	0.07
**Caesarean N(%);**			
Glargine and Lispro	11(42%)	15 (57%)	0.07
CSII	8(42%)	11(56%)	0.8
Premixed human insulin	4(50%)	4(50%)	0.5
Detemir and Aspart	0	2(66%)	0.9
NPH and insulin analogue	4(40%)	6(60%)	0.1
**Non elective Caesarean N(%);**			
Glargine and Lispro	6(33%)	12 (66%)	0.06
CSII	5(38%)	11(62%)	0.09
Premixed human insulin	2(40%)	3(60%)	0.5
Detemir and Aspart	0	0	
NPH and insulin analogue	3(40%)	5(60%)	0.08

Values are given as mean +/− SD or number of subjects (n) with percentage in parenthesis.

Forty-nine (48%) of pregnant women scheduled the gestation. The association of PCC and obstetrics and perinatal outcomes was studied. Only the gestational age at birth was statistically significant with respect PCC (P value = 0.04).

With regard to progression of maternal microvascular complications during pregnancy, four patients (2.9%) had progression of nephropathy and twenty-three (16.3%) had progression of retinopathy. The association of different insulin therapies and progression of maternal microvascular complications were not significant (results not shown).

## Discussion

The goal of glycaemic control in pregnancy is to reach a value of Hab1c as close to the normal range, without hypoglycaemia^1^. In order to achieve this goal, in addition to complying with adequate hygienic and dietary habits, an adjustment of the insulin treatment, using the model of insulin therapy in an individualised way to the characteristics of each patient, is required^6^. Our patients had a mean HbA1c of 6.53 +/− 1.05%; we observed an improvement of the metabolic control throughout the gestation, regardless of the type of insulin treatment. Our data confirm other reports^10^.

Observational studies show that an HbA1c greater than 1% of the normal range is associated with a higher rate of congenital anomalies and miscarriages with respect to the general population^12^. The rate of miscarriages in our series was 7.3%. In other series with scheduled pregnancies it was 5.7%^13^ and with unscheduled pregnancies from 14%^3^. As we have already mentioned, all miscarriages occurred in patients with 10 or more years of duration of DM and poor metabolic control. Results confirm other studies that have shown that poor metabolic control in early gestation, especially during embryogenesis, is related to a higher incidence of miscarriages and congenital anomalies^14,15^. With respect to fetal deaths, we had 3,6%. Results confirm other studies^12^.

The pre-pregnancy HbA1c of our pregnant women was 7.3 +/− 1.58%, higher levels than those recommended^5^, which coincides with the fact that there were a small number of scheduled pregnancies. It is clear that obtaining pre-pregnancy optimum metabolic control is of great importance. However, only 28% of pregnant women scheduled their gestation. In other series 75% of gestations were unscheduled^12^. This could be attributed to the fact that in our study we also evaluated patients with type 2 PGDM, who may have had less knowledge about their disease and the impact on gestation. Pre-pregnancy glycaemic control plays a fundamental role in reducing the frequency of fetal outcomes. When we analysed the relationship between pre-pregnancy HbA1c and obstetrics and perinatal outcomes, we found weight percentile, end of pregnancy route and gestational age at birth were statistically significant. Therefore, a better pre-pregnancy metabolic control improves the incidence of preterm delivery, which is consistent with what has already been published^16^.

The majority of our patients with type 2 PGDM received insulin treatment with short-acting insulin analogues and NPH or premixed human insulin, while our patients with type 1 DMPG received treatment with MDI (glargine and lispro more frequently) or CSII. Currently there are many options for insulin treatment in pregnant patients. Until now the recommended treatment has been short-acting insulin analogues and NPH^17^. After recognizing the safety and efficacy of insulin analogues^9^, it is possible to treat with long-acting analogues, especially if there is a risk of nocturnal hypoglycaemia^18,19^. However, there are no studies that clearly demonstrate the benefits over the rest of insulin therapies and extensive clinical experience with long-acting analogues is required^20,21^. With respect to treatment with CSII, some studies have specifically compared treatment with MDI vs. CSII^22,23^. There are studies where CSII therapy was more effective in improving the mean HbA1c and glycaemia than MDI therapy^23,24^. But in more recent studies, although mean blood glucose was lower with CSII, no differences were observed with respect to HbA1c^25^. In our study, there were no differences with respect to metabolic control among patients treated with MDI or CSII. In a recent Cochrane review^26^, it was concluded that there is no evidence that show that the use of one particular form of insulin administration is better than another in pregnant women with diabetes.

Poor metabolic control during gestation is associated with a higher prevalence of macrosomia^4^. In our study, the lower the mean HbA1c during pregnancy, the lower the percentage of caesarean sections, macrosomia, abortions and fetal deaths. Data confirm what is described in the literature^4,27^.

We also analysed the insulin therapeutic modality used, with the progression of microvascular maternal complications during pregnancy. In our study we observed a progression of retinopathy of 16.3%, a percentage similar to other studies that have shown a progression of diabetic retinopathy between 20 and 55%^28^. However, this is not easily comparable because it would be necessary to differentiate type 1 from type 2 DM, in order to affirm a smaller progression of complications in our series. In diabetic nephropathy, there are no conclusive studies in this respect. In our patients we observed a progression of nephropathy of 2.9%. 14.5% had microalbuminuria and 6.9% had nephropathy before gestation. These data coincide with what has been published, with a prevalence of approximately 11% of microalbuminuria and 5% of nephropathy^29^. Two large studies of women with type 1 diabetes mellitus, DCCT (Diabetes Control and Complication Trial) and the PCS (EURODIAB Prospective Complications Trial), concluded that gestation is not a risk factor for the development of retinopathy, nephropathy or early neuropathy after correction of confounding factors such as age, duration of diabetes and HbA1c^28^.

These results are limited and still inconclusive because the study compares the effectiveness of different insulin therapies in patients with PGDM and does not discriminate subcategories of PGDM (type 1 and type 2 DM). In addition well-designed randomised clinical trials are needed to draw a conclusion.

## Conclusion

The different insulin therapies used in patients with PGDM does not seem to affect obstetric and fetal outcomes. Poor glycaemic control prior to and during gestation is associated with poor obstetric-fetal outcomes. Patients who have PPC have a lower HbA1c and a reduction of obstetric and fetal outcomes. Further specifically designed studies are needed to evaluate metabolic, obstetric and fetal outcomes depending on different insulin treatment used.
